# Difference-making factors in implementing a quality improvement program for sleep apnea in stroke/TIA patients

**DOI:** 10.1186/s43058-026-00944-9

**Published:** 2026-04-17

**Authors:** Nicholas A. Rattray, Edward J. Miech, Dawn M. Bravata, K. Maya Story, Laura J. Myers, Brian B. Koo, Laura Burrone, Ali Sexson, Stanley E. Taylor, Anthony J. Perkins, Joanne Daggy, Jason J. Sico

**Affiliations:** 1 Department of Veterans Affairs (VA) Health Systems Research (HSR) Expanding Expertise Through E-health Network Development (EXTEND) Quality Enhancement Research Initiative (QUERI), Indianapolis, IN USA; 2 VA HSR Center for Health Information and Communication (CHIC), Richard L. Roudebush VA Medical Center, HSR&D Mail Code 11H, 1481 West 10 Street, Indianapolis, IN 46202 USA; 3 Regenstrief Institute, Indianapolis, IN USA; 4 Department of Medicine, Indiana University School of Medicine, Indianapolis, IN USA; 5 Medicine Service, Richard L. Roudebush VA Medical Center, Indianapolis, IN USA; 6 Department of Neurology, Indiana University School of Medicine, Indianapolis, IN USA; 7 Department of Biostatistics and Health Data Science, Indiana University School of Medicine, Indianapolis, IN USA; 8 Neurology Service, VA Connecticut Healthcare System, West Haven, CT USA; 9 Pain Research, Informatics, and Multi-Morbidities, and Education (PRIME) Center, VA Connecticut Healthcare System, West Haven, CT USA; 10 Department of Neurology, Yale School of Medicine, New Haven, CT USA; 11 Department of Internal Medicine, Yale School of Medicine, New Haven, CT USA

**Keywords:** Mixed methods, Configurational comparative methods, Local context, Intervention fit, Evaluation, Quality improvement, Stroke, Obstructive sleep apnea

## Abstract

**Background:**

Effective prevention of ischemic stroke and transient ischemic attack (TIA) involves timely, guideline-concordant risk factor management. Obstructive sleep apnea (OSA), a significant but underdiagnosed cerebrovascular risk factor, affects approximately 70% of stroke and TIA patients. Untreated OSA is linked to impaired post-stroke recovery, recurrent vascular events, and increased mortality. Despite guideline recommendations to consider early post-stroke/TIA OSA screening, few patients receive sleep studies. This study explores the implementation of a multidisciplinary quality improvement intervention for OSA management at six Department of Veterans Affairs medical centers between 2021 and 2024, focusing on contextual factors influencing implementation success.

**Methods:**

This mixed-methods study used data from the Addressing Sleep Apnea Post-Stroke/TIA (ASAP) stepped-wedge cluster-randomized clinical trial (NCT04322162). We conducted qualitative analyses of provider interviews and quantitative assessments via configurational comparative methods (CCMs) to identify difference-making conditions for successful implementation. The Group Organization (GO) score, a facility-level measure indicating team cohesion and activation in diagnosing and treating OSA among patients with acute cerebrovascular events, served as the primary implementation outcome.

**Results:**

Successful implementation, defined by a GO score of ≥ 6, was achieved at four of the six facilities. Four conditions were sufficient by themselves for implementation success: implementation of sleep test ordering, monitoring sleep testing processes, post-discharge care coordination, and positive influence of champions during implementation.

**Conclusions:**

This study highlights the interplay between local context and novel clinical practices in successful program implementation of an acute sleep service. Four difference-makers perfectly distinguished between sites with and without implementation success. These findings provide actionable insights for tailoring and timing implementation strategies to improve adoption.

**Trial registration:**

ClinicalTrials.gov NCT04322162.

**Supplementary Information:**

The online version contains supplementary material available at 10.1186/s43058-026-00944-9.

Contributions to the literature

- Identifies Mechanistic Pathways for Normalization: Demonstrates how four sufficient conditions (sleep test ordering, monitoring of sleep testing processes, post-discharge care coordination, champion influence) function as Normalization Process Theory mechanisms—collective action and cognitive participation—through which organizational elasticity enables complex clinical innovations to become routine practice.
- Advances Normalization Process Theory: We provide empirical evidence that organizational elasticity determines whether sites can implement the NPT mechanisms required for normalization. Our findings explain why sites with favorable contextual factors (positive CFIR scores, motivated champions) still fail without sufficient elasticity, demonstrating that context alone does not guarantee normalization; organizational capacity to enact change mechanisms is critical.
- Clinical Practice Translation: We offer insights into practical strategies for implementing acute sleep services post-stroke/TIA. Configurational analysis reveals multiple, diverse difference-makers for normalization, which suggests the importance of enabling healthcare systems to tailor implementation processes to their local organizational context when bridging inpatient cerebrovascular care with outpatient sleep medicine.

## Introduction

Effective prevention of stroke and transient ischemic attack (TIA) involves guideline-concordant vascular risk factor management delivered in a timely manner [1]. Obstructive sleep apnea (OSA) has been identified as an underdiagnosed and inadequately treated cerebrovascular risk factor [2] and occurs in approximately 70% of patients with stroke/TIA [3–8]. Impaired post-stroke recovery, increased recurrent vascular events, and higher mortality rates have been associated with untreated OSA [9–11]. Existing clinical guidelines recommend considering early post-stroke/TIA OSA screening and treatment, if present. Although over 11,000 Veteran patients with ischemic stroke or TIA present at Department of Veterans Affairs (VA) medical centers annually, few patients receive OSA testing [12, 13].

Studies suggest the importance of timely identification, testing, and treatment for OSA among patients with cerebrovascular disease [14]. However, the broader question of how to translate the guideline recommendations into routine practice remains less explored. Due to variation in the organization of healthcare delivery systems, understanding the interplay between local context at individual hospitals and the implementation of novel clinical practices is needed. In the Veteran Health Administration (VA)—the largest integrated healthcare system in the United States (US)—medical centers vary in their ability to implement new programs that involve multiple services (i.e., hospital medicine, sleep medicine, nursing, neurology), especially those spanning inpatient and outpatient settings. Further contributing to the complexity of developing, implementing, evaluating, and refining cross-service implementation activities are local practices. For example, a pronounced challenge in launching a new acute sleep medicine program is that standard sleep medicine services are almost exclusively delivered in outpatient settings, whereas acute cerebrovascular care occurs in Emergency Departments and inpatient settings.

Researchers have argued for increased attention to use of theories in implementation science, including developing micro-theories that help explain potential causal mechanisms [15]. A deeper understanding of mechanisms may aid in determining how, when, and where to pinpoint opportunities to translate research into clinical practice, to build theory-driven explanations that can be adapted to different contexts, and provide direction to healthcare operations personnel regarding what resources would be most necessary to successfully implement innovative healthcare delivery programs [16–18]. May and colleagues’ Normalization Process Theory (NPT) proposes that successful implementation occurs when innovations become embedded into routine practice through collective action by human agents within specific contexts [19, 20]. NPT posits that normalization, or the process by which innovations become routine, depends critically on organizational elasticity: the capacity and flexibility within healthcare settings to absorb, implement, and sustain new practices [21]. When elasticity is sufficient, organizations can build the infrastructure and coordinate the activities necessary for interventions to become “business as usual.” NPT identifies collective action (the operational work of implementation) and cognitive participation (sustained commitment and engagement) as key mechanisms through which innovations become normalized into routine practice. In this analysis, we examine how organizational elasticity—the capacity to absorb, coordinate, and sustain new practices—enables sites to implement the collective action and cognitive participation mechanisms central to normalization, measured by the GO score as a proxy for team cohesion and routine embedding.

This analysis examines the dynamic role played by contextual factors from a clinical trial focused on sleep apnea testing in patients admitted to a hospital for an acute ischemic stroke or TIA. We conducted an implementation evaluation from the Addressing Sleep Apnea Post-Stroke/TIA (ASAP) clinical trial (NCT04322162). ASAP was designed to implement a quality improvement intervention to increase the assessment and management of OSA among stroke/TIA patients through an implementation trial at six diverse medical centers. Our research question was: what contextual factors were difference-makers for successful implementation and normalization of the ASAP intervention? We hypothesized that organizational elasticity would determine whether sites could implement the processes necessary to embed acute sleep services into routine practice.

The ASAP intervention successfully increased 30-day OSA diagnostic testing from 2.1% at baseline to 29.1% during active implementation, demonstrating that implementation is feasible [22]. However, the question of how and why implementation succeeded at some sites but not others remained unexplored. To answer this question, we used configurational comparative methods (CCMs). CCMs offer a case-based approach that uses applied set theory and Boolean algebra to determine the crucial set of difference-making factors. Rather than assessing the incremental effect of a unit difference in an independent variable on a dependent outcome while controlling for all other variables, CCMs identify key factors that are necessary or sufficient for an outcome to occur [23, 24]. We used CCMs to determine the particular bundles of implementation strategies and local contextual factors that together determined successful normalization of acute sleep services..

## Methods

### Design and parent study

This study used a mixed methods study design. Specifically, we analyzed transcripts using a deductive-inductive approach and conducted multi-value configurational analysis on the basis of scored implementation factors and validated quantitative instruments. For the qualitative analysis, we grounded the study in the Consolidated Criteria for Reporting Qualitative Research [25]. Reporting on configurational analyses adhered to current recommendations [24, 26].

This study was part of ASAP, a stepped-wedge cluster-randomized clinical trial conducted from May 2019 to January 2024. Full details of the ASAP trial, including rationale for site selection, consent procedures, and data validation approaches are available [27]. The six implementation sites were selected from ten VA facilities with the highest annual volumes of ischemic stroke/TIA admissions; four declined and six agreed to participate. The six participating sites were randomly assigned to one of three waves (2 sites per wave). Wave 1, active implementation, occurred Feb 2021-Nov 2022; Wave 2 was Sept 2021-Jun 2023; and Wave 3 was Apr 2022-Jan 2024. The 21-month active implementation data were collected in three 7-month increments for each of the waves; we refer to the first 7-month increment for each site as Period A, the second as Period B, and the third as Period C. The intervention at the site level was considered quality improvement and a waiver of informed consent was obtained from the VA Institutional Review Board (IRB) for patients and staff as the research involved minimal risk. A HIPAA waiver was obtained from the VA IRB to identify patients and collect medical record data [27].

### Description of ASAP

ASAP was a multi-component quality improvement intervention designed to increase OSA testing and treatment among stroke/TIA patients [22, 27]. The intervention was based on systems redesign principals and included: a virtual kickoff during which site teams identified improvement opportunities, brainstormed solutions and developed a site specific action plan; a virtual collaborative; electronic health record (EHR) tools (e.g., to identify potentially eligible patients); a web-based platform to store and display data, action plans, and a library of resources (e.g., guidelines, educational materials). The implementation strategies included external facilitation by study staff to site team members and audit and feedback. All sites received the ASAP intervention, as well as external facilitation and audit and feedback. Our configurational analysis examines the contextual factors and local adaptations that enabled successful implementation and normalization of this intervention at some sites but not others. Each facility was resourced to allocate up to 0.5 full-time equivalent funding for a field staff member. Local site investigators received five hours per week of protected time throughout active implementation.

### Study participants and data collection

Several sources of primary data were used: qualitative, categorical, and quantitative. The analytic team collected data from a purposive sample of health providers at the six participating VA hospitals, which was expanded through snowball sampling to include all relevant health practitioners involved in the QI project. Clinical providers involved in cerebrovascular care or sleep medicine volunteered to participate in the local quality improvement project with the intended goal to create an acute inpatient sleep service for Veterans with a recent cerebrovascular event. Interviews were conducted before and after the active implementation period. In addition, monthly assessment data and self-reported progress were recorded by study team members at collaborative calls.

### Data sources and collection

The Consolidated Framework for Implementation Research (CFIR) [28] guided the overall evaluation of contextual factors in the ASAP intervention (Table 1). Primary qualitative data were collected from participants using questionnaires (e.g., monthly progress report in Research Electronic Data Capture [REDCap]) [29] and interactions through direct contact with facility staff recorded with fieldnotes. Formal interviews occurred at baseline and at the end of active implementation. Interviews were conducted through video calls with key informants from inpatient (e.g., neurologists, hospitalists) and outpatient (e.g., sleep medicine providers) providers. Interviews were audio recorded, transcribed verbatim, and de-identified. NVivo 12. [30] was used for data management and coding. Using deductive code from CFIR 1.0 and emergent codes from a first round of open coding, codebooks were developed. Code books were initially created for baseline and revised slightly at end of active implementation interviews based on a round of open coding (see Appendix A). Qualitative analyses involved two teams of two qualitative researchers independently reading and re-reading each transcript, assigning labels and codes to data segments, and addressing discrepancies through discussion. The codebook was tested and refined by applying codes to exemplar transcripts. A second round of focused coding ensued with two separate analysts applying codes to each transcript. The process was completed separately for baseline (36 codes) and end of active implementation (25 codes) transcripts. We used the constant comparison technique [31] to identify emerging themes in the data, including special attention to themes related to context (e.g., champions), processes created and refined in the creation of an acute sleep service, external facilitation, and audit and feedback. Analytic memos were used to connect emergent themes and facilitate discussions among the analytic team [32].

**Table 1:** Factors, source, and values

Factors	Data Source	Categories and Calibration	
Consolidated Framework for Implementation Research (CFIR) Constructs • Planning • Champions • Goals & Feedback • Reflecting & Evaluating • Champions • Networks and Communications • External Policies • Evidence Strength	Study Team voting	−2	Strong negative
		−1	Moderate negative
		0	Minimally referenced
		+ 1	Moderate positive
		+ 2	Strong positive
Acute Sleep Service Processes of Care • Identifying Patients • Tracking Patients • Order Testing • Treating Sleep Apnea	Observational, Site Self-Report	1	Minimal: not performing the process or doing so seldomly
		2	Partial: carrying out the process with some inconsistency
		3	Full: performing the process consistently
Quality Dashboard Usage Data	Hub Dashboard Reporting	1	Low (< 10 users/month)
		2	High (≥ 10 users/month)
Local Site Investigators • Pulmonology vs Neurology • Sleep vs Stroke • Responsibilities Changed During Study • Research Experience • Quality Improvement Experience	Observational, Site Self-Report	0	Dichotomous values for presence or absence of certain categories
		1	
Implementation Barriers • Identifying Patients • Tracking Patients • Ordering Sleep Studies • Completing Sleep Studies • Treating Patients • Other Barriers	Site Self-Report	1–3	Low
		4–9	Medium
		≥ 10	High
External Facilitation Dose	Facilitation Tracking	< 10	Low
		10–20	Medium
		> 20	High
Diagnostic Strategy	Administrative, Observational, Site Self-Report	1	HST* used on inpatients
		2	Expedited outpatient testing
		3	Combination of inpatient HST and expedited outpatient testing

^*^HST refers to home sleep test

To aggregate information from the individual level to the medical center level, the study team used a real-time, all-digital secret ballot process to assign scores at the site level for CFIR construct magnitude (i.e., positive, neutral, negative or minimally referenced) and valence (i.e., weak or strong). The evaluation team voted on each CFIR construct for each of the six sites at each of the three time points (i.e., data periods A, B, and C). Final ratings for each CFIR construct required 80% agreement or higher [33, 34]. The analytic team tracked a variety of factors including facility and provider characteristics and participant behavior using REDCap and structured note templates. Factors analyzed in this configurational study were selected a priori based on CFIR constructs and implementation science literature, hypothesized to be causally relevant to implementation success and normalization. The frequency that participants used a quality dashboard that monitored facility performance was reviewed and summarized for analysis. “Implementation Activities” were defined as discrete activities conducted by participating sites to implement an acute sleep service. The evaluation team tracked the implementation activities, grouped by categories: clinical program, EHR/clinical informatics, professional education, patient and caregiver education, quality improvement, data feedback, professional development, and other implementation activities. Barriers to implementation were assessed across six domains related to the process of testing and treating patients OSA: (1) identifying patients eligible to offer testing to, (2) tracking patients during the hospitalization period and post-discharge, (3) ordering testing, (4) completing testing once ordered, (5) treating patients found to have OSA, and (6) other barriers.

### Outcome

The Group Organization (GO) score for diagnosing and treating sleep apnea among patients with stroke or TIA was the primary implementation outcome [35, 36]. The GO score served as our measure of implementation success, distinct from the clinical effectiveness outcomes (30-day diagnostic testing rates, treatment rates) reported in the primary trial. Building on prior studies [33, 36], the GO score captured the degree to which sites developed sustainable systems and team capacity i.e., the extent of normalization rather than simply temporary compliance with the intervention (see Appendix B). It indicated the degree of team cohesion and activation to provide sleep apnea management among cerebrovascular disease patients, and has been used in prior stroke-related studies. The evaluation team independently determined each facility's GO score through triangulation of multiple data sources: interview data, monthly assessment data, and self-reported progress on implementation activities during collaborative calls [27]. Team cohesion reflected the degree to which inpatient and outpatient services (hospital medicine, neurology, sleep medicine) coordinated to provide sleep apnea care. Team activation indicated the extent to which teams had built routine processes for identifying eligible patients, ordering tests, and ensuring treatment. The GO score uses a 1–10 scale. In this analysis, implementation success was designated as a final GO score of 6 or greater.

### Configurational analysis

CCMs draw on concepts from set theory, formal logic and Boolean algebra to identify the crucial difference-making conditions for an outcome of interest among a defined set of cases [37, 38]. CCMs focus on cases, conditions, and combinations rather than variables, and answers research questions like: "What made a difference, for whom, and under what conditions?” Within the broader family of CCM approaches, this study conducted the configurational analysis using Coincidence Analysis (CNA), which offers a systematic approach to identify difference-makers with small sample sizes [24, 39, 40].

Furthermore, output from CNA analyses is at the level of conditions (i.e., factor values), rendering it well-suited for our analysis in this study given that our potential explanatory conditions consisted of ordinal factors. For example, as described earlier the ratings assigned to individual CFIR constructs ranged from +2 (strong positive influence) to −2 (strong negative influence). The particular numbers used in the CFIR construct scores, however, only serve as “markers” or “tags” for the underlying qualitative concepts they represent, and thus have an ordinal, not interval, relationship to one another. While the +2 to −2 construct scoring approach we used is conventional within implementation science [41, 42], we could have easily used different numbers to represent the same fundamental concepts.

CNA, on the other hand, is custom-designed to analyze this kind of data, applying a bottom-up algorithm and a systematic routine to search for difference-makers at the level of factor values [40, 43]. As CNA is a case-based method, the six VA medical centers served as the cases in this study. Implementation scientists have found CNA to be an approach particularly well-suited for examining real-world complexities inherent within the field, with over 30 articles appearing in the peer-reviewed implementation science literature since 2020. The CNA package for the R environment (“cna”) was used to conduct the CCM analyses, along with the software applications R and R Studio [44].

### Factor calibration

To develop the analytic dataset for the configurational analysis, we drew on factors collected during the active implementation period of the ASAP trial. Factors were calibrated after data collection was complete. The calibration approach is described in Table 1. Some factors were categorized as dichotomous (presence/absence), whereas multi-value ordinal factors were categorized using levels (e.g., high, medium, low; strong positive influence to strong negative influence, etc.).

### Data reduction

To reduce our data in the first stage of exploratory analysis, we used a common procedure in CNA studies called the “msc” routine to scan the entire original dataset to identify factors with particularly strong connections to the outcome; this procedure has been described in detail in prior literature [45, 46]. In order to remain consistent within our overall CCMs approach, we used a data reduction strategy that operated entirely within a “regularity theory” framework rather than applying other well-established methods used in statistics because those methods use linear algebra (as opposed to Boolean algebra), focus on associations at the variable level instead of at the factor value level, and operate within a fundamentally different “interventionist” framework [40].

Two parameters of fit were used: consistency and coverage. Consistency is a measure of reliability and represents how often CNA results correctly identify cases where the outcome of interest is present. For example, if a difference-maker identified by CNA covered three cases where the outcome was present but also one case where the outcome was absent, the consistency score would be 75% (3/4). Coverage is a measure of explanatory breadth and represents how many cases are accounted for by CNA results. For instance, if a difference-maker identified by CNA accounted for 9 out of 10 cases with the outcome of interest, the coverage score would be 90%. The “msc” routine comprehensively and exhaustively examined every 1-, 2-, and 3-condition configurations instantiated in the dataset, checked to see if each configuration met a given consistency threshold, retained those that did, and organized the results into a condition table for review. [40, 46] For example, clinical lead background and training were examined as potential conditions in the configurational analysis but did not meet sufficiency criteria and are therefore not reported as difference-makers. The output from the msc routine also allowed us to identify key threshold levels that mattered within individual factors and then dichotomize factors around those thresholds [47]. For example, when only the maximum value for a factor met the 100% consistency level, we dichotomized around that maximum value (e.g., if Sleep Test Ordering = Full, then the metafactor for Sleep Test Ordering was assigned a value of 1; all else was assigned a value of 0); when adjacent values within a factor both met the 100% consistency level (e.g., + 2 and +1 values for Champions), we dichotomized around the lower of the adjacent values (e.g., if Champions +1 or +2, then the metafactor for Champions was assigned a value of 1; all else was assigned a value of 0). Using this process, we identified a set of four difference-making factors that met all of the following four criteria: 100% consistency; 100% coverage; consistent with theory and background knowledge; and relevant to the research question [47].

## Results

### Facility characteristics

As shown in Table 2, the six participating VA hospitals were spread throughout US, with half within the southern US region known as the “Stroke Belt.” [48]. Thirty-seven interviews were conducted at baseline; 21 were completed at the end of active implementation. Implementation teams included a physician lead (neurologist, pulmonologist, or internal medicine) and field staff (with research or clinical backgrounds).

**Table 2:** ASAP facility characteristics by GO score

Characteristic	Facility					
	A	B	C	D	E	F
Interviews: baseline, end of active implementation	7,3	3,3	9,6	8,6	4,2	6,1
Lead Clinical Background	Pulmonology	Neurology	Pulmonology	Neurology	Internal Medicine	Neurology
Trained in Sleep Medicine	Yes	Yes	Yes	Yes	Yes	No
Trained in Vascular Neurology	Yes	Yes	No	No	No	Yes
Field Staff Background (number)	Research (1)	Clinical (1)	Clinical (2)	Research (1)	Research (2)	Clinical (1)
Geographic Region	Midwest	Southwest	Southwest	South	Southeast	South
Diagnostic Testing Level^a^						
Period A	Low	Medium	High	Low	Low	Low
Period B	Medium	Medium	High	Low	Medium	High
Period C	High	High	High	Medium	Medium	High
GO Score^b^						
Period A	4	4	5	2	4	5
Period B	5	5	7	4	4	6
Period C (the primary implementation outcome)	8	7	9	4	5	8

^a^The diagnostic testing level was defined as the receipt of any sleep study (attended or unattended, in-laboratory, or in-home) within 30 days of admission for the index stroke or transient ischemic attack (TIA). Sites were classified as: low (< 20% of eligible patients received testing), medium (20%–39%), and high (> 40%). The 21-month active implementation data were collected in three 7-month increments; the first 7-month increment was Period A, the second 7-month increment was Period B, and the third 7-month increment was Period C

^b^The Group Organization (GO) score for diagnosing and treating sleep apnea among patients with stroke or TIA was the primary implementation outcome and indicated the degree of team cohesion and activation to provide sleep apnea management among cerebrovascular disease patients

### Difference-makers

**Fig. 1:**
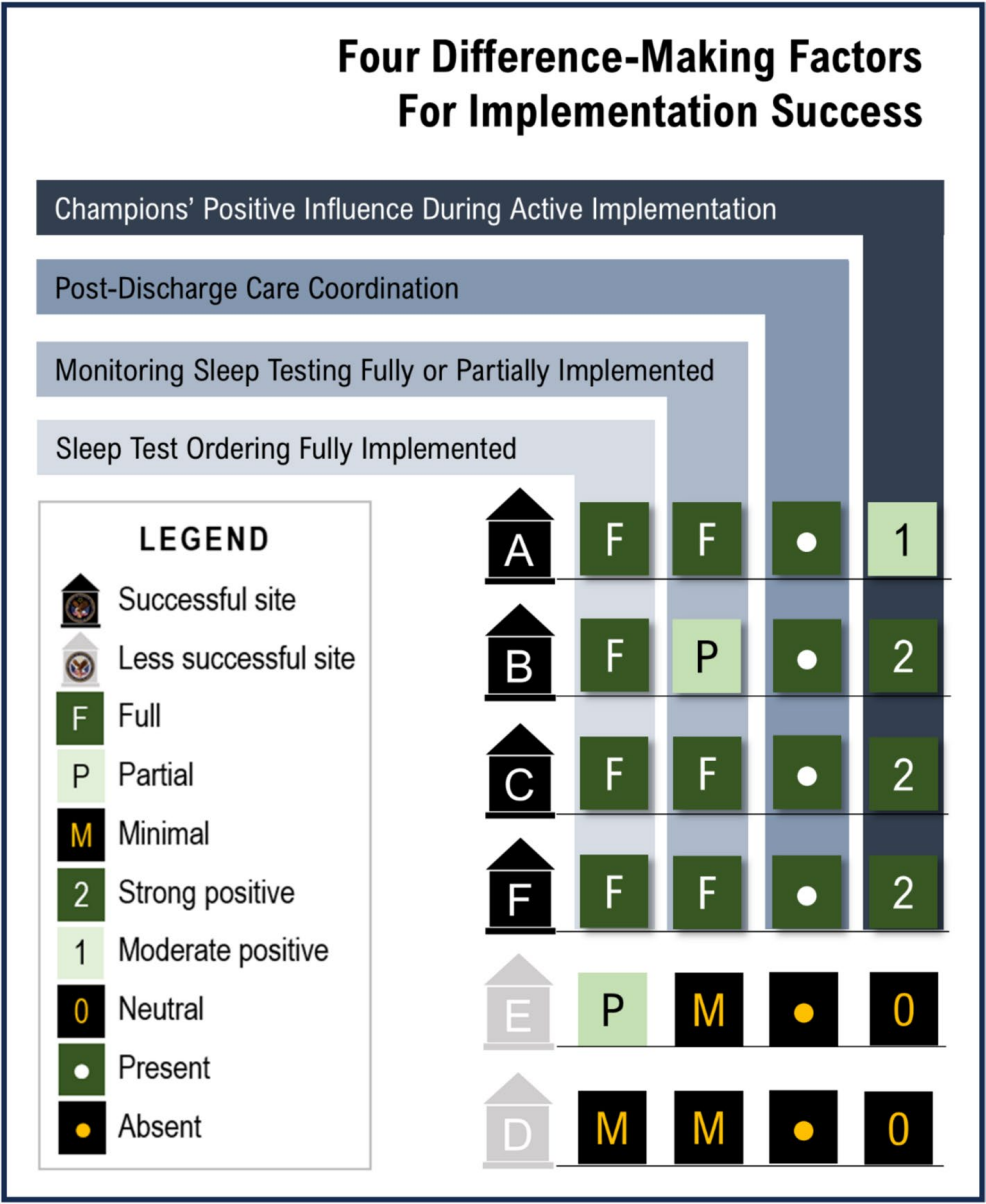
Four difference-making factors for implementation success

Four difference-makers were sufficient by themselves for implementation success, distinguishing between those that achieved a GO Score of ≥ 6 from those that did not. Three of these related to the extent to which processes of care measures were in place (sleep test ordering, monitoring sleep testing processes, post-discharge care coordination) and one related to champions having a positive influence during active implementation; Fig. 1 and Table 3). Each of these four difference-making factors yielded the outcome with 100% consistency and 100% coverage.

**Table 3:** Qualitative evidence illustrating difference-making factors

*Difference-Maker 1* Sleep Test Ordering	Description: System for ordering tests for patients who have been identified as eligible for testing by their clinical team and who have agreed to be tested. Testing could be ordered by a clinician, most often by a member of the inpatient care team Specific activities included: (1) Creation of new or modification to existing electronic health record (EHR) orders sets and workflow, and; (2) orientation of clinical providers within and across service lines to new tools and workflow which support sleep test ordering
*Example Activity*	*Exemplar Quotation or Field Observation*
EHR modifications and changes in workflow implemented	“Getting neurology on board was pretty instrumental in that for us because [t]hat suggestion to get OSA testing is built into their note now, and it’s just going to continue to be something that the hospitalists and care teams can order.” (Interview, Site A)
	A Veteran admitted with either stroke/TIA is eligible for ASAP, [MD 2] adds a note via CPRS and cosigns for [MD 1] for review. [MD 1] reviews and lets [MD 2] know if the patient is willing/eligible for ASAP and will order consult for Home Sleep Test set up, and alerts [MD 2] for HST set up. (Observation, Site B)
Difference-Maker 2 Monitoring Sleep Testing	Description: System developed to monitor and coordinate activities related to successful completion of sleep testing during the cerebrovascular event hospitalization period. Includes coordinating tasks related to ordering and successful completion and interpretation of sleep study performed during the hospitalization period Specific activities included: (1) system created to monitor status of sleep testing completion, which includes testing being performed, results interpreted, and results made available to clinical team; and (2) ASAP team member coordinates and enhances cooperation between inpatient (i.e., hospitalists, neurologists, nursing) and outpatient (i.e., sleep medicine personnel) clinical and administrative teams and facilitating completion and interpretation of sleep study findings during the hospitalization period
*Example Activity*	*Exemplar Quotation or Field Observation*
Field Staff Monitoring Patients Inpatient stay	“We can get people set up and started really quickly; whereas in most private sector situations or at least the ones that I worked in in the private sector, the DME [durable medical equipment] is sort of removed. That we’re in a lucky spot where we can just walk to the patient’s room and put them on the device that they’re going to go home … I do keep track of them through looking daily at a particular ward through CPRS [electronic medical record]. I can tell if there is a new patient admitted. (Site A)
Coordinating across services for Home Sleep Testing prior to Hospital Discharge	[ASAP MD] spoke with the physician (at post-discharge rehabilitation center) and arranged for nurse to pick up a home sleep study from sleep disorders center and return the unit the following day. [MD] reviewed the study, and the patient had AHI of 16.8/Hour. (Observation, Site B)
Communication across services to coordinate delivery of sleep care	Respiratory Technician at the VA CPAP clinic, is going above and beyond to help with CPAP set-ups while patients are still in the hospital. This has ensured that newly diagnosed OSA patients are able to receive testing and treatment prior to discharge and do not need to wait on the lengthy CPAP clinic outpatient scheduling/appointment processes. (Observation, Site C)
Difference-Maker 3 –Post-Discharge Care Coordination	Description: System developed to monitor successful completion of sleep testing post-discharge. ASAP Field staff continues care coordination initiatives and communication related to sleep care others. Communication may be initiated by the ASAP Field Staff member, outpatient personnel who care for post-stroke/TIA patients, or patients, and care partners Specific activities included: (1) monitoring that sleep testing not completed during the hospitalization period is arranged and completed after discharge; (2) communicating with outpatient clinicians (e.g., respiratory technicians, sleep physicians, neurologists) and other key stakeholders. Topics of communication may include those related to obtaining sleep testing not performed during the hospitalization period and questions related to appointments, trouble-shooting sleep testing equipment concerns, and CPAP therapy
*Example Activity*	*Exemplar Quotation or Field Observation*
Coordinating across services for Home Sleep Testing prior to Hospital Discharge	“We created a different flow sheet–-If a patient is gone and they’ve been discharged and they are diagnosed, if the patient needs to get set up with PAP, what steps are we going to take and how are we going to inform people. So, really just getting a good pathway, which is nice. I have relayed our pathway multiple times.” (Site A)
Serves as a Care Navigator between Sleep Medicine service and patients after hospital discharge	“I [ASAP Field Staff] was the one doing the calls to outpatients. Like hey, you may be interested in getting tested. Our sleep team is stellar and also very, very stretched thin.” (Site A)
*Difference-Maker 4* Positive Influence of Champions During Active Implementation	Description: The Champion team at each site was at minimum dyadic, consisting of at least one Clinical Champion and one Field Staff. Champions had to have a positive influence on successful implementing an acute sleep service by the end of active implementation Specific activities included: (1) communicating about the benefit of OSA testing and treatment soon after a cerebrovascular event to patients, care partners, and other providers; (2) creating/modifying tools and workflows for an acute sleep medicine service, and; (3) liaising between services which provide cerebrovascular and sleep care
*Example Activity*	*Exemplar Quotation or Field Observation*
Clinical Champion displaying atypical dedication to patient care and ensuring implementation occurs	“You’re already overworked. We’re short staffed. No one told her about it. it’s all worked out nicely, and she’s like super gung-ho about it. She’s a neurologist as well as a sleep medicine physician. She understands the importance of the treatment and everything too–-We’ll have like nurses or something that don’t know. They really don’t know, and so I’ll just explain it to them. They’re like oh, really? This is interesting. Wow. I didn't know that. So, your kind of explaining all of these things, the correlation between stroke and sleep apnea and things like that. Because really, it’s all about education.” (Site B)
Working in tandem and communicate with other healthcare providers regarding the importance of sleep apnea testing among stroke/TIA patients	“So, finding them [ASAP Field Staff], So, I’d be in charge of identifying them, and that could be sometimes days later [after discharge], and then [Local Site PI] was reaching out to their primary care team and saying hey (laughter). So, we noticed that your patient had a stroke. Here is why we’re doing this. So just like a constant process of explaining why we were doing what we were doing because there are so many hospitalists and just like various staff members that would have been in charge of putting in those consults.” (Site A)
Champion demonstrates processes needed to order sleep testing to peers	“[OUR CHAMPION] is someone who’s already like doing it, right? Like if you have one nurse … who can show the other nurses like hey, this is what we do. It’s really easy, it’s great, it takes five minutes and they get all this information. It’s just like hey, this is what we’re doing—I can help you set it up on that patient, that type of thing. I think that really helps. (Site C)

In Table 3, we describe the individual difference-makers, with examples of discrete activities involved in each. Exemplar quotations or field observations illustrate how each of these activities occurred at specific sites.

#### Explaining difference-makers from providers’ perspectives

Developing a system for timely ordering sleep tests during the hospitalization (Difference-Maker #1) and monitoring the status of testing from the time that they are ordered to being completed (Difference-Maker #2), as well as robust staff involvement in post-discharge care coordination (Difference-Maker #3) were key difference-makers that were directly linked to implementation success during the active implementation period. The fourth difference-maker involved active involvement of champions during the active implementation period. Reviewing specific cases illustrates how the difference-makers distinguished more successful sites from less successful sites. At facility C, the implementation champion laid out a goal of “not just being a Sleep Lab but becoming a Sleep Center.” Articulating and implementing such a philosophical approach led to meaningful multidisciplinary communication; this site developed capacity to order sleep tests on patients identified as being eligible for and receptive toward testing, and to track patients’ sleep care status:

Our physicians [gave] us permissions to utilize our day home sleep study team as well as our night team, registered sleep technologists. [We had] had back-ups to back-ups. If we knew that certain team members maybe could not perform setting up a unit, we might have our day team go ahead and present it upstairs and apply it on the patient... and tried to adjust to another night. We did call upstairs to see if the patient was gonna be discharged early, or we made sure that we had open communication with the floors to make sure we didn’t miss a patient.” (Site C)

Facility A likewise fully implemented elements of an acute sleep service in part through making sleep apnea testing routine. Although this facility had originally targeted hospitalists, they found that involving other personnel such as field staff to assist with ensuring tests were ordered, completed, and results made available to all parties involved in active care management, proved essential for successful implementation.

Field staff with clinical or research backgrounds were involved in several distinct activities. Yet the activities that distinguished sites with higher success were field staff involvement in coordinating patients during inpatient stays in post-discharge care. Each of those conditions were independently sufficient to achieve success. A physician described how field staff played a key role in coordination of care between inpatient stroke management:

the resource needed is actually on-the ground manpower, and so [Field Staff] fits into it … they’re not actually setting up the device themselves. They’re going to be communicating back and forth—delivering or expediting certain processes. (Site A)

This site champion viewed the role of field staff as overseeing patient care to ensure that consults were acted on, which at this facility, involved “an immediate turnaround CPAP study” or an MD evaluation for more complex patients.

The third difference-maker featured a time-dependent condition. At the final data period of active implementation (i.e., Period C—active implementation months 15–21), facilities where champions had a positive influence achieved success. At one successful site, the champion described themselves performing key tasks:

When the patient needs to be set up, I set up the patient … I download everything. Send it to [MD]. She puts the results in there. I call the patient. Set up the patient on CPAP. (Site B)

As outlined in Table 3, other key clinical champion tasks (Difference-Maker #4) included being proactive in identifying patients who may be eligible for sleep testing, discussing these patients with appropriate healthcare team members across inpatient and outpatient settings, and educating colleagues about the rationale and importance of an acute sleep service for stroke and TIA patients.

#### Less successful sites

Further insights can be gained from examining sites that did not achieve ≥ 6 GO scores At Site E, implementation factors (generally positive scores for champions, reflecting & evaluating, networks & communication, evidence strength) suggested a promising environment for implementation. However, this facility faced important barriers that hindered its ability to implement ASAP.

First, the clinical champion described coordination challenges, such as the geographic barrier of three miles between the sleep clinic and the main hospital: *“when we [have] two separate facilities … it just presented a new challenge in a VA because then there is an absence of ability to interact with patients on the inpatient side of things—Just the scheduling, it becomes another barrier for Veterans to have to wait to get care.” (Site E).*

The sleep clinic also faced a staff shortage and lack of “buy-in” from the nursing service, and by the end of active implementation, had not been able to address these challenges. A second issue for this facility was the inability to fully implement components of an acute sleep service, namely a method for identifying and tracking patients. Both facilities with less successful implementation were unique in lacking the capacity to track patients. A third issue specific to this facility was turnover in leadership (i.e., service chiefs and chief of staff) that impacted capacity and morale during the active implementation period. Finally, the local team at Site E reported a higher level of uncertainty in the early phase of implementation, as one interviewee explained, *“I know that there is a stroke team in place, and it’s unclear to me exactly what’s the specific pathway that they fall under. Are they just consultative? Are they contacted in the emergency room? Are they contacted on the floor?”* Despite some favorable contextual factors, the inability to develop a system for tracking patients and ordering tests, as well as insufficient involvement of field staff, led to only partial implementation of an acute sleep service for patients with cerebrovascular disease.

At Site D, the inability of champions to secure alignment with the sleep medicine lab led to lower implementation success. As one interviewee described, “*I would try to get the night techs to help out, and they were like it’s not our job to do that and things like that.”* Both sites D and E were stymied by difficulties gaining support from nursing or sleep medicine.

## Discussion

In this study, we considered a range of potentially important contextual factors in developing novel acute sleep services at six medical centers. Implementation success across the ASAP intervention sites varied substantially (GO scores 4–9), with four sites achieving normalization (GO scores ≥ 6) and two sites experiencing only partial implementation despite favorable conditions. This variation occurred despite all sites receiving identical intervention components and external facilitation support, underscoring the critical role of organizational elasticity in translating intervention exposure into normalized practice. We used configurational analyses to identify difference-makers that consistently differentiated the more successful four facilities from the two less successful facilities. Difference-makers linked to greater success included full implementation of sleep test ordering system, full or partial implementation of a system for monitoring patients for testing, active involvement of field staff in post-discharge care coordination, and the positive influence of champions at the active phase of implementation.

Proposing an “acute sleep service” may be seen by some as running contrary to traditional divisions of labor between inpatient cerebrovascular care services (typically provided by neurologists, hospitalists, and inpatient nursing) and outpatient sleep medicine. Study participants disclosed in interviews how ASAP represented fundamentally new approaches to testing for OSA, which aligns with stroke patients’ perspectives on sleep testing [49].

Healthcare facilities seeking to implement acute sleep services for the care of patients with stroke or other conditions (e.g., post-surgical, right heart failure) should consider the following lessons learned: infrastructure is needed to track the target population, processes for ordering sleep studies (especially while patients were still hospitalized) should be implemented, care coordination is required to ensure findings and tasks are not dropped during the transition from inpatient to outpatient settings, and clinical champions play a critical role in supporting the establishment of robust systems that become embedded within the routine functioning of the facility. Implementation sites were resourced to allocate up to 0.5 full-time equivalent funding for field staff [27] For scale-up beyond research settings, organizations would need to determine whether to create dedicated care coordinator positions or redistribute these responsibilities among existing staff. Our findings suggest that without dedicated field staff for care coordination (Difference-Maker 3), normalization is unlikely to occur even with favorable contextual conditions.

### Contribution to implementation science

This study contributes empirical evidence demonstrating how organizational elasticity enables the normalization of complex clinical innovations. Our findings extend Normalization Process Theory by identifying the specific manifestations of elasticity, operationalized through four difference-making factors, that distinguished sites where acute sleep services became embedded in routine practice from those where implementation remained incomplete. The GO score, which measures team cohesion and activation to provide sleep apnea care, served as an indicator of the degree to which the intervention transitioned from a temporary project to "business as usual." Higher GO scores (≥ 6) reflected organizations where testing and treating OSA in stroke/TIA patients had become more routine rather than exceptional. Our configurational analysis revealed how the four difference-makers map onto key NPT constructs necessary for normalization.

Critically, sites with lower GO scores (< 6) lacked organizational elasticity to implement these difference-makers despite possessing some favorable contextual factors (positive CFIR scores, motivated individuals). Sites D and E faced barriers—geographic separation between services, staff shortages, inability to secure cross-service buy-in—that constrained their capacity to build the necessary infrastructure and sustain the collective action required for normalization. This finding underscores that elasticity is not merely about having resources, but about the organizational capacity to marshal, coordinate, and sustain those resources in service of embedding new practices. These results shed light on moving from implementation to normalization in complex healthcare environments requiring cross-service collaboration. Rather than identifying individual factors as universally important, our configurational approach revealed that any one of the four difference-makers was sufficient for success—suggesting multiple pathways to normalization when elasticity is present. For organizations implementing similar innovations, these findings suggest that prioritizing infrastructure development (monitoring systems, care coordination roles, workflow integration) and maintaining champion engagement throughout implementation are critical investments for achieving normalization, not merely initial adoption.

Although this study offers insights into difference-makers in implementation, we describe certain limitations from the research design and methods. The configurational analysis relied on nominal or ordinal values for explanatory factors and outcomes that represented underlying qualitative concepts (e.g., levels), which means that determining appropriate parameters for classification required decisions based on clinical expertise and field observations. The study was conducted in six VA medical centers—the relatively small sample size may limit the generalizability to other healthcare settings or populations and internal validity given the constraints on detecting potential additional configurations or relationships between those configurations. While the prevalence of the outcome of interest was moderately high at 66.7% (i.e., present in 4 out of 6 cases), the use of consistency and coverage thresholds of 100% allowed for identification of bona fide difference-makers. Factors that did not appear in the configurational results should not automatically be deemed as unimportant: even though they do not meet the formal definition of difference-makers, they may contribute in other ways to successful implementation. Heterogeneity in site-specific adoption, while necessary, introduced variability that may have affected the consistency of results. The study spanned several years, during which there were significant external factors (e.g., COVID-19 pandemic, global recall of sleep apnea machines) that could have impacted implementation and outcomes, complicating the attribution of success to specific interventions [50]. Any of the four key difference-makers were sufficient by themselves for perfectly distinguishing cases with and without implementation success; we reported all four because they provided complementary insights. Some data were self-reported by sites, which may have introduced reporting biases.

## Conclusion

This study offers an analysis of factors that made a difference in the successful implementation of sleep apnea management in stroke/TIA patients at six VA hospitals, highlighting the importance of implementing processes for sleep test ordering, monitoring sleep testing processes, post-discharge care coordination, and the influence of champions during active implementation. Successful sites developed systems for patient tracking and sleep test ordering. This study contributes to understanding the interplay of process and contextual factors in implementing clinical innovations and offers insights for improving outcomes in other conditions that require cross-service collaboration.

## Supplementary Information

Additional file 1. Appendix A. Baseline Interview codebook.

Additional file 2. Appendix B. Supplement_GO Score_ASAP.

## Data Availability

The data that support the findings of this study must remain on Department of Veterans Affairs servers. Please contact the corresponding author if you are interested in working with these data.

## Abbreviations

TIA: Transient ischemic attack
OSA: Obstructive sleep apnea
CCMs: Configurational comparative methods
GO score: Group Organization
VHA/VA: Veteran Health Administration
US: United States
CFIR: Consolidated Framework for Implementation Research
EHR: Electronic health record
REDCap: Research Electronic Data Capture
CNA: Coincidence Analysis
