# Supplementary material for: Difference-making factors in implementing a quality improvement program for sleep apnea in stroke/TIA patients
Source: Implement Sci Commun. 2026 Apr 17;7:109. doi: 10.1186/s43058-026-00944-9 (PMC13224654; doi:10.1186/s43058-026-00944-9)
Supplement: Supplementary file 1 — Additional file 1. Appendix A. Baseline Interview codebook. [file 43058_2026_944_MOESM1_ESM.docx]

Appendix A: Codebook for Baseline Interviews (edited for clarity)

|  | Code | Description | Exclusion/Inclusion |
| --- | --- | --- | --- |
| 1 | 100_Strengths_OSA-related | Refers to strengths related to getting patients tested and treated for OSA. This may include having such resources as staffing and supplies, infrastructure, administrative support, and geographic localization of sleep services. | Includes: strengths identified by subjects and those identified by coders.  Excludes: general comments about sleep infrastructure that do not have a valence. |
| 2 | 110_Barriers_OSA-related | Refers to barriers related to getting patients tested and treated for OSA. This may include having such resources as staffing and supplies, infrastructure, administrative support, and geographic localization of sleep services. | Includes: weaknesses identified by subjects and those identified by coders.  Excludes: general comments about sleep infrastructure that do not have a valence. |
| 3 | 120_Future resources_how directed_OSA-related | Refers to how sites would use future resources to support the mission of ASAP in getting patients tested and treated for OSA. | Includes: How sites would use resources that come with ASAP (i.e., 0.5 FTE field staff) and what resources may be needed in the future to develop and maintain ASAP.  Excludes: |
| 4 | 130_Coordination Across Services-OSA related | Refers to how coordination occurs across services to have patients tested for OSA. | Includes: Outpatient or inpatient care coordination with the sleep service.  Excludes: Coordination of stroke and TIA patients that does not involve OSA (e.g., involved hypertension management). |
| 5 | 140_Future data reports_preferences_priorities_OSA-related | Refers to how they either currently receive or would like to receive data reports regarding OSA testing and treatment. | Includes: Reports generated about sleep study access and/or backlog.  Excludes: Comments about data reports unrelated to OSA (e.g., ED flow). |
| 6 | 180_Sleep Medicine Infrastructure | Refers to the infrastructure in place to have patients tested and treated for sleep problems, and specifically for OSA. | Includes: This may include staffing (e.g., including sleep fellows), supplies, sleep testing equipment, administrative support, standard operating procedures, the availability of sleep services (e.g., e-consults, inpatient consults).  Excludes: Stroke/TIA infrastructure apart from sleep services (e.g., carotid ultrasound clinic). |
| 7 | 190_Other comments_OSA-related | Items that seem relevant to OSA testing and treatment but do not fit into categories 100 to 180. | Includes:  Excludes: Comments not related to being tested or treated for OSA. These may include sleep services for other types of sleep disorders (e.g., insomnia). Comments that fall into categories 100 to 180. |
| 8 | 200_Experiences_data_documentation_Stroke | Refers to experience with documentation on stroke and/or TIA patients. | Includes: Progress notes, reporting performance measures (e.g., documenting the NIHSS), discharge documentation.  Excludes: Non-stroke-TIA conditions (e.g., heart failure patients, ED patients without stroke/TIA). |
| 9 | 210_Experience_virtual community_General | Refers to experience that subjects have participating in other virtual communities that promote information exchange, collaboration, education, and dissemination of learning and best practices. | Includes: Maybe referred to as communities of practice. May be within the VA or outside of the VA (e.g., those with professional organizations).  Excludes: Non-stroke-TIA conditions (e.g., heart failure patients, ED patients without stroke/TIA). |

| 10 | 300_How ASAP aligns w other org goals | Refers to how improving the timeliness and OSA testing and treatment done within ASAP aligns with other goals within the organization. Organizational goals will most likely be at the level of the VAMC but can extend beyond the single VAMC to broader levels within VHA (e.g., VISN) or outside of VHA (e.g., national societies). | Includes: If ASAP is aligned or not with organizational goals. Subjects may report that parts of ASAP do and do not align with local VAMC goals.  Excludes: Other programs unrelated to ASAP and their alignment with organizational goals (e.g., a telehealth program without a sleep component to it aligning with local VAMC goals). |
| --- | --- | --- | --- |
| 11 | 310_Leadership_how important is ASAP | Specifically refers to how important ASAP is to VAMC leadership. | Includes: Leadership within the clinic, the service line(s), and the hospital. May also include VA Central Office leadership.  Excludes: Leadership at university affiliate. |
| 12 | 320_Degree of confidence in ASAP success | Refers to the degree of confidence the subject has in the success of ASAP. They may report that some parts may be more successful than others (e.g., testing more than treating). | Includes: The degree of confidence that they report and that others involved in ASAP report.  Excludes: Success in unrelated stroke/TIA initiatives or non-ASAP sleep initiatives. |
| 13 | 330_Motivation to participate in ASAP | Refers to their motivation to be part of ASAP. | Includes: The subject’s motivation or the motivation of others in their work unit (e.g., sleep lab).  Excludes: Motivation NOT to participate. |
| 14 | 400_Formal policy or protocol_OSA in Stroke-TIA | Whether the sites have preexisting protocols about testing and treating OSA among stroke/TIA patients. | Includes:  Excludes: Formal policy or protocols for OSA testing and treatment that are more general (i.e., any Veteran) or more specific to another disease state (e.g., heart failure). Also excludes order sets. |
| 15 | 410_Coordination w Neurology_OSA in Stroke-TIA | How OSA testing and treatment for stroke/TIA patients is coordinated with the Neurology service. | Includes: Items like how patients are referred from Neurology to Sleep Clinic for OSA in either inpatient or outpatient/clinic settings.  Excludes: Coordination of OSA testing and treatment for stroke/TIA patients with other services (e.g., Primary Care, Emergency Medicine). |
| 16 | 420_Order sets_menus_PSG_PAP | Do sites report having order sets related to sleep studies and OSA treatment. | Includes: Order sets where providers can order outpatient and inpatient sleep consults, consults for the sleep lab, eConsults, PAP therapy, etc.  Excludes: Order sets for services not related to OSA testing and treatment. |
| 17 | 430_How well diagnosed-treated_OSA in Stroke-TIA | Refers to the degree to which patients with ischemic stroke and TIA are currently getting tested and treated for OSA. | Includes: This may include their experience of getting patients tested and treated for OSA as well as their sharing that these patients are historically not identified as having OSA.  Excludes: Non stroke/TIA diagnoses. |
| 18 | 450_Identifying patients_OSA in Stroke-TIA | Refers to how patients are identified as having a stroke or TIA. | Includes:  Excludes: |
| 19 | 460_Owning patients_OSA in Stroke-TIA | Identifies as who is responsible the different parts of OSA care among stroke/TIA patients. Different services may have different roles/responsibilities (e.g., the primary care provider or the hospitalist “owns” ordering the testing whereas the sleep service “owns” getting the test done and treating patients). | Includes: Who is responsible for ordering the testing, getting the testing done, acting on a test that is positive for OSA, and then treating patients once OSA is found.  Excludes: Non OSA-related testing. |
| 20 | 470_3 most influential people_OSA in Stroke-TIA | Who subjects identify as the most important people in getting patients tested and treated for OSA. | Includes: Specific individuals/titles within their own VAMC or VISN.  Excludes: Those at other VAMCs. |
| 21 | 480_Resource constraints_OSA in Stroke-TIA | Resource constraints that exist in terms of getting patients tested and/or treated. Discussion of this may also come up as sites review their sleep infrastructure. | Includes: Resources include staffing and supplies, infrastructure, administrative support, and geographic localization of sleep services.  Excludes: Resource constraints of the entire VAMC or VHA unless it applies to OSA (e.g., PAP supply reduced because of Respironics recall). |
| 22 | 600_Stroke team | Members of the stroke team. This is commonly discussed in the context of stroke protocols. | Includes: Inpatient and outpatient providers that constitute the stroke team. This is largely looked like inpatient providers that may admit and/or consult on patients admitted with an ischemic stroke and to a lesser extent TIA. They may be Emergency Medicine providers, nurses, Neurologists, Hospitalists, and could be attendings, fellows, or residents.  Excludes: Rapid Response Team that responds to non-stroke codes. |
| 23 | 610_Admission_Stroke-TIA | Who is the primary team who admits a stroke/TIA patient into the hospital. This is commonly discussed with order sets. | Includes: Typically either Neurology or Internal Medicine. Never sleep. Can also include admission under an “observation” status.  Excludes: Non stroke-TIA diagnoses. |
| 24 | 620_Experiences_data_documentation_OSA-related | Refers to experience with documentation on OSA. | Includes: Progress notes, reporting performance measure, entering documentation related to sleep studies. This is not specific to stroke/TIA and can refer to documentation related to OSA for any type of patient.  Excludes: Non OSA sleep conditions (e.g., insomnia). |
| 25 | 630_In-hospital management_Stroke-TIA | Describes what is done for the Veteran admitted with stroke/TIA, including testing performed and medication management. | Includes: Testing and therapies received in the hospital.  Excludes: Outpatient management. |
| 26 | 640_Post-discharge outpatient follow-up_Stroke-TIA | Describes the post-discharge follow-up that is arranged by the in-hospital team. | Includes: Outpatient appointments made with sleep, primary care, neurology.  Excludes: Information related to readmissions or subsequent Emergency Department visits. |
| 27 | 660_Order sets_menus_Stroke-TIA | Describes the order sets that are used by the primary team to admit patients with a stroke or TIA. This is commonly discussed with admissions. | Includes: CPRS order sets  Excludes: Sleep order sets (unless the sleep order set is incorporated into the stroke/TIA order set). |
| 28 | 700_Last Stroke-TIA patient | Refers to the experience of caring for their most recent stroke/TIA patient. | Includes: Inpatient or outpatient care of a stroke/TIA patient.  Excludes: Non stroke/TIA patients. |
| 29 | 800_Staff education_General | Refers to how staff are educated about new policies, procedures, as well as how they receive continuing medical education. This is not specific to stroke/TIA or OSA. | Includes: Education provided within their VAMC.  Excludes: Education provided outside of VHA (e.g., professional organizations). |
| 30 | 810_Learning Climate_General | The overall learning climate within their VAMC. Does learning/in-servicing happen often and is it supported? Are sites open to learning new things? | Includes: In-servicing, formal lectures, lunch lectures, delivered in person or virtually.  Excludes: Learning climate outside of VHA. |
| 31 | 815_Experiences_data_documentation_General | Refers to experience with any type of medical record documentation. | Includes: Progress notes, reporting performance measure, entering documentation related to the provision of healthcare. This can be inpatient or outpatient.  Excludes: Non-medical record documentation. |
| 32 | 817_Experience with QI_General | Refers to experience within VHA regarding performing quality improvement (QI) work. QI work does not have to be specific to a disease state nor does it have to be specific to healthcare delivery (e.g., may be related to workflow in the cafeteria). | Includes: Any experience with QI within VHA.  Excludes: QI experience outside of VHA. |
| 33 | 820_Interact beyond home VA_General | Refers to how subjects interact outside of their own VAMC. | Includes: Interactions can occur outside of their own VAMC but within VHA (e.g., between VAMCs, within the VISN, with VA Central Office, with National Sleep Program). It can also occur with professional societies outside of VHA and with academic affiliates or private practice.  Excludes: Interactions within their home VAMC (e.g., across service lines). |
| 34 | 830_Culture | Refers to the norms, values, and basic assumptions of a given organization. Specifically refers to the culture that is their current state. | Includes: The culture of the service line (e.g., sleep) or within the broader VAMC.  Excludes: The culture that they would like to develop. |
| 35 | 900_Quotable Quotes | Things you really liked. | Includes: Quotes that are paper worthy.  Excludes: Quotes you would never want to see in a paper. |
