# Supplementary material for: Difference-making factors in implementing a quality improvement program for sleep apnea in stroke/TIA patients
Source: Implement Sci Commun. 2026 Apr 17;7:109. doi: 10.1186/s43058-026-00944-9 (PMC13224654; doi:10.1186/s43058-026-00944-9)
Supplement: Supplementary file 2 — Additional file 2. Appendix B. Supplement_GO Score_ASAP. [file 43058_2026_944_MOESM2_ESM.docx]

**Group Organization (GO) Score – Providing ASAP Care**

| Mastery  10 | **ASAP SYSTEM**  **ASAP Practices Defined by**  **Facility-Wide**  **ASAP System** | **Nationally Recognized For Excellence in Providing ASAP Care.** Facility widely known and recognized for sustained system of excellence in ASAP-specific practices. |
| --- | --- | --- |
| Advanced Proficiency  9 |  | **ASAP System Plus Real-Time Monitoring.** ASAP practices defined by mature facility-wide multidisciplinary ASAP system plus real-time or near-real-time monitoring of ASAP patients by ASAP system staff during patients in hospital stay. ASAP system staff respond and/or intervene as indicated to meet patient needs and/or meet expectations of ASAP system of care (e.g., data or documentation missing from medical record, existing data indicate patient requires timely follow-up, etc.) ASAP system staff can intervene appropriately by sending email messages, making phone calls, having in-person conversations, accessing medical records, providing care themselves, etc.) Excellence in ASAP-specific practices a clear priority of facility. New ASAP-specific practices and innovations routinely trialed. |
| Intermediate Proficiency  8 |  | **ASAP System of Care.** ASAP practices defined by comprehensive, 24/7 facility-wide multidisciplinary ASAP system of care that is mature, appropriately supported with part- and/or full-time positions, firmly rooted within larger organization, and can handle the departure of key personnel. ASAP system staff actively support and promote facility-wide ASAP program. ASAP system has its own identity largely independent of traditional organizational silos. ASAP-specific practices have largely been mastered throughout facility and constitute a full-fledged system of ASAP care. |
| Basic Proficiency  7 | **ASAP PROGRAM**  **ASAP Practices Defined Primarily**  **by Facility-Wide ASAP Program** | **ASAP Practices Defined by Facility-Wide ASAP Program, 24/7.** ASAP practices defined primarily by comprehensive facility-wide ASAP program rather than traditional organizational silos or individual provider preferences. ASAP protocols and procedures implemented, with formal collaboration mechanisms in place and used routinely. Proficient ASAP care consistently provided throughout facility on 24/7 basis, and cross-service ASAP-specific practices coordinated across all areas and all shifts. Providers feel sense of responsibility and/or accountability for ASAP care outside of their own immediate area. |
| Emerging Proficiency  6 |  | **ASAP Practices Defined by Facility-Wide ASAP Program, Regular Business Hours.** ASAP practices defined primarily by comprehensive facility-wide ASAP program rather than traditional organizational silos or individual provider preferences. ASAP protocols and procedures implemented, with formal collaboration mechanisms in place and used routinely. During regular business hours, proficient ASAP care consistently provided throughout facility and cross-service ASAP-specific practices coordinated across all areas. Providers feel sense of responsibility and/or accountability for ASAP care outside of their own immediate area. |
| Advanced Developing  5 | **ASAP COMPONENTS**  **Cross-Service Practices Emerging Specific to ASAP;**  **Developing Facility-Wide ASAP Approach** | **Implementation of Facility-Wide Approach Specific to ASAP**. Providers from different clinical areas coordinating cross-service practices related to components of ASAP-specific care. Cross-service communication and collaboration around ASAP starting to happen routinely. Basic ASAP protocols and procedures have been developed and formally approved, and their implementation has begun. Variation in ASAP practices based on individual providers within services and units not a major issue. |
| Developing  4 |  | **ASAP Practices Influenced By Cross-Service Processes, with Development of Facility-Wide Approach Specific to ASAP.** Multidisciplinary group starting to develop a facility-wide approach specific to ASAP that formally coordinates cross-service ASAP-specific care and transcends traditional organizational silos. ASAP-specific protocols, procedures, templates, collaboration mechanisms, etc. at least partially developed. ASAP practices vary based on shift and clinical area. |
| Advanced Beginning  3 | **PEOPLE**  **ASAP Practices Defined by Traditional Organizational Silos and Individual Providers;**  **No Facility-Wide Approach to ASAP** | **ASAP Practices Dominated By Organizational Silos and Individuals, with Some** **Collaboration Around Specific ASAP practices.** Traditional silos of units, departments, and services dominating ASAP practices. Clusters of providers within and/or across departments and services starting to communicate and collaborate on semi-regular basis around particular ASAP-specific practices and initiatives. No multidisciplinary ASAP team or program. ASAP practices vary based on shift and clinical area. No facility-wide approach specific to ASAP. |
| Beginning  2 |  | **ASAP Practices Defined By Organizational Silos and Individuals, with Some** **Individual-Driven ASAP Activity**. Individuals practicing on their own within traditional silos of units, departments, and services. Cross-service ASAP-specific practice and communication occurring on limited, ad hoc basis based on individual providers involved. Certain individuals pursuing ASAP-specific practice-based initiatives based on own priorities and areas of perceived need. No mechanisms designated specifically for ASAP-specific cross-service communication. No special procedures for responding to in hospital ASAPs. No multidisciplinary ASAP team or program. ASAP practices vary based on shift and clinical area. No facility-wide approach specific to ASAP. |
| 1 |  | **ASAP Practices Defined By Organizational Silos and Individuals.** Individuals practicing on their own within traditional silos of units, departments, and services, with no sense of responsibility or accountability for ASAP care outside their area. Little to no activity, communication, or collaboration happening related specifically to ASAP care. No multidisciplinary ASAP program. Substantial variation in ASAP practices within services and units based on individual providers. No mechanisms designated specifically for cross-service communication for ASAP patients. No special procedures for responding to in hospital ASAPs. No facility-wide approach specific to ASAP. |

Note: The evaluation team met to discuss each facility's GO score across the three time periods (A, B, C) and voted on the score. Although the ASAP analysis plan specified ≥80% agreement among team members, nearly all scores across time periods were achieved by unanimous team opinion, reflecting strong consensus on facility performance
